# Supplementary material for: SPONGE: simple prior omics network GEnerator
Source: Bioinformatics. 2025 Jun 27;41(7):btaf320. doi: 10.1093/bioinformatics/btaf320 (PMC12964359; doi:10.1093/bioinformatics/btaf320)
Supplement: btaf320_Supplementary_Data [file btaf320_supplementary_data.zip › SPONGE_SD.pdf]

Supplementary data for:

SPONGE: Simple Prior Omics Network GEnerator

The role of prior networks

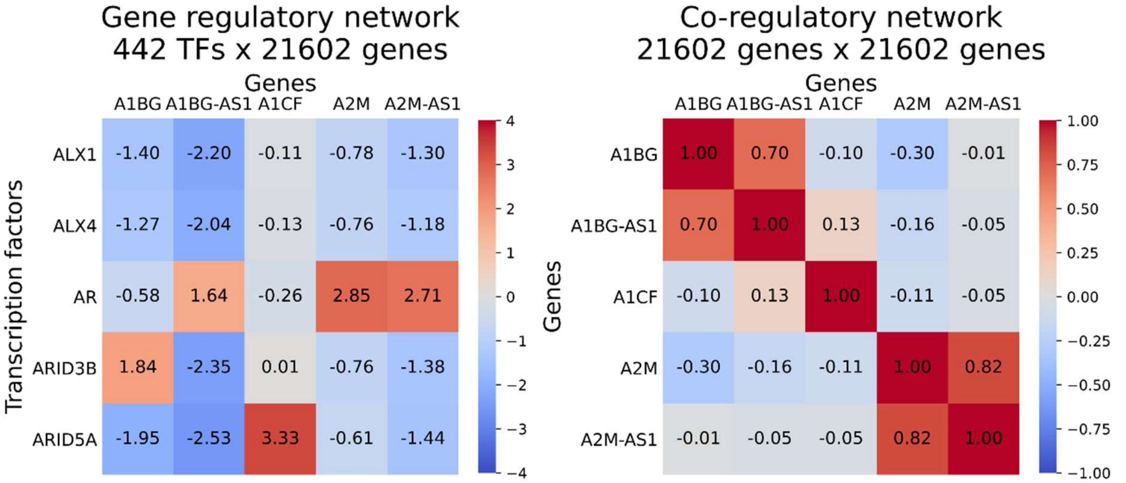

Fig. S1: The comparison between two different networks created with the inclusion of prior networks (left) and without them (right). For visualization purposes, only the first five rows and columns are shown.

When the PANDA algorithm[1] is used to generate gene regulatory networks, it relies on three sources of information: the prior gene regulatory network linking transcription factors to genes, the prior protein-protein interaction network describing interaction between the transcription factors, and finally gene expression data for multiple samples. In theory, the prior networks can be omitted. However, the network being generated then is a different type of network—a co-regulatory network which describes associations between genes. Regulatory and co-regulatory networks are not directly comparable, owing to their different dimensions (N(TF) x N(gene) vs N(gene) x N(gene), see Fig. S1) and biological interpretation.

Gene regulatory networks are bipartite, directly describe regulatory relationships, and the interpretation of an edge would be the proposed strength of regulation. Co-regulatory networks, on the other hand, should be interpreted only as the likelihood of genes being co-regulated. This also changes the type of analysis that can be performed: estimating the overall strength of regulation for a gene would be difficult from a co-regulatory network, but simple for a gene regulatory one.

To summarise, the addition of a gene regulatory prior network into the PANDA algorithm allows us to refine our initial estimate with the gene expression data to generate a final consensus network. Without this information, a very different network would be created, less suitable to answer the research questions that interest us.

The comparison with earlier prior networks

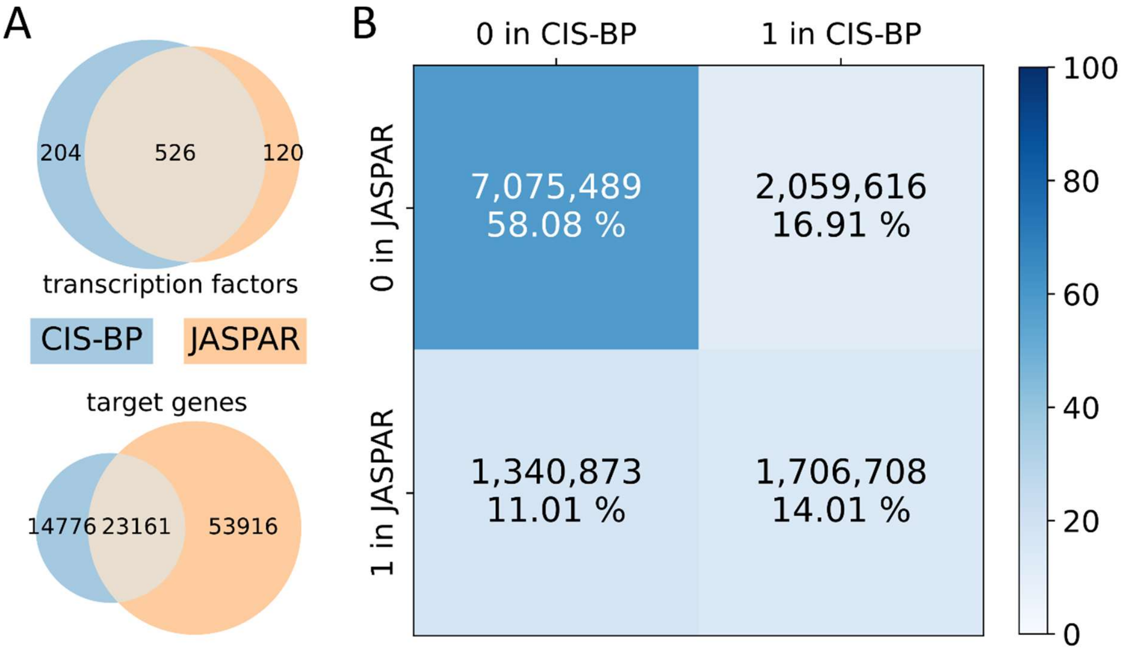

Fig. S2: The comparison between the prior networks generated previously using CIS-BP motifs and the ones generated with SPONGE (JASPAR motifs). A) The overlap between the transcription factors and genes captured in the two networks. **B)** The correspondence between the assigned edges for the transcription factors and genes common to both prior networks.

To determine how our prior network generation approach compares to the ones used previously, we have compared the SPONGE network to one generated previously using the FIMO[2] tool in the MEME[3] suite using motifs from the CIS-BP[4] database. This prior was also designed primarily for use with PANDA. In Fig. S2, we have summarised the comparison.

In Fig. S2A, the correspondence between the transcription factors and genes present in the two networks is shown. Most transcription factors overlap, with minor differences possibly due to naming conventions, the fact that the older prior includes non-human vertebrate transcription factors, and that they come from CIS-BP[4] rather than JASPAR[5]. The overlap for genes is lower, but again we believe this to mostly be because of the naming mismatch, as both priors are based on the same gene annotations from UCSC[6].

In Fig. S2B, we show the confusion matrix for the two priors, similar to Fig. 1E in the main text. It is restricted only to the transcription factors and genes common to both priors. This time, we see that the match between the two priors is much worse, which can indicate differences such as filtering thresholds and the mismatch between a binding site score as calculated by JASPAR and the p-values calculated by FIMO. The agreement is higher on the negative edges but given that they represent the majority of possible edges this is hardly surprising. The overall number of positive edges is very similar.

References

1. Glass, K., Huttenhower, C., Quackenbush, J. & Yuan, G. C. Passing Messages between Biological Networks to Refine Predicted Interactions. *PLoS One* **8**, (2013).

2. Grant, C. E., Bailey, T. L. & Noble, W. S. FIMO: Scanning for occurrences of a given motif. *Bioinformatics* **27**, 1017–1018 (2011).

3. Bailey, T. L., Johnson, J., Grant, C. E. & Noble, W. S. The MEME Suite. *Nucleic Acids Res.* **43**, W39–W49 (2015).

4. Weirauch, M. T. *et al.* Determination and Inference of Eukaryotic Transcription Factor Sequence Specificity. *Cell* **158**, 1431–1443 (2014).

5. Rauluseviciute, I. *et al.* JASPAR 2024: 20th anniversary of the open-access database of transcription factor binding profiles. *Nucleic Acids Res.* **52**, D174–D182 (2024).

6. Perez, G. *et al.* The UCSC Genome Browser database: 2025 update. *Nucleic Acids Res.* **53**, D1243–D1249 (2025).
