# Supplementary figures and images for: SPONGE: simple prior omics network GEnerator
Source: Bioinformatics. 2025 Jun 27;41(7):btaf320. doi: 10.1093/bioinformatics/btaf320 (PMC12964359; doi:10.1093/bioinformatics/btaf320)

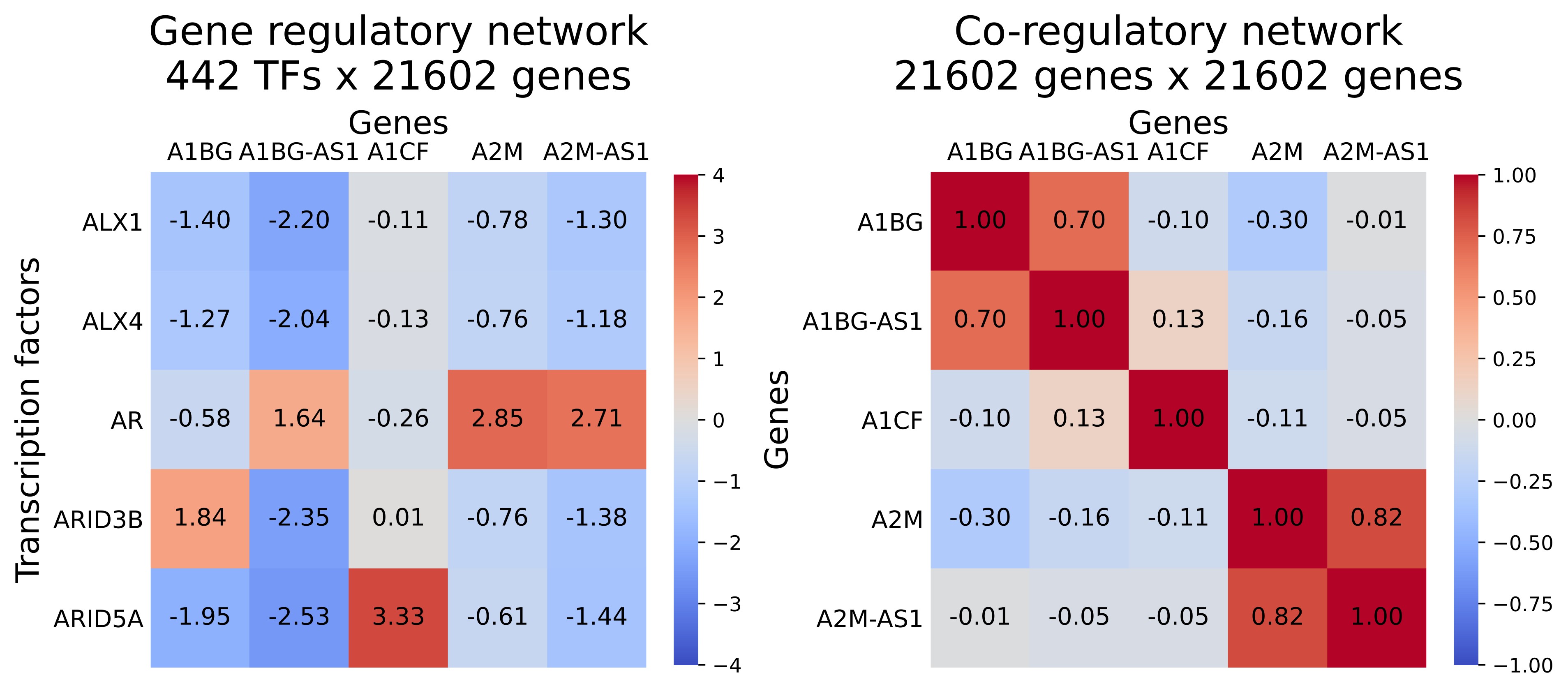

Supplement: btaf320_Supplementary_Data [file btaf320_supplementary_data.zip › figure_s1.jpg]

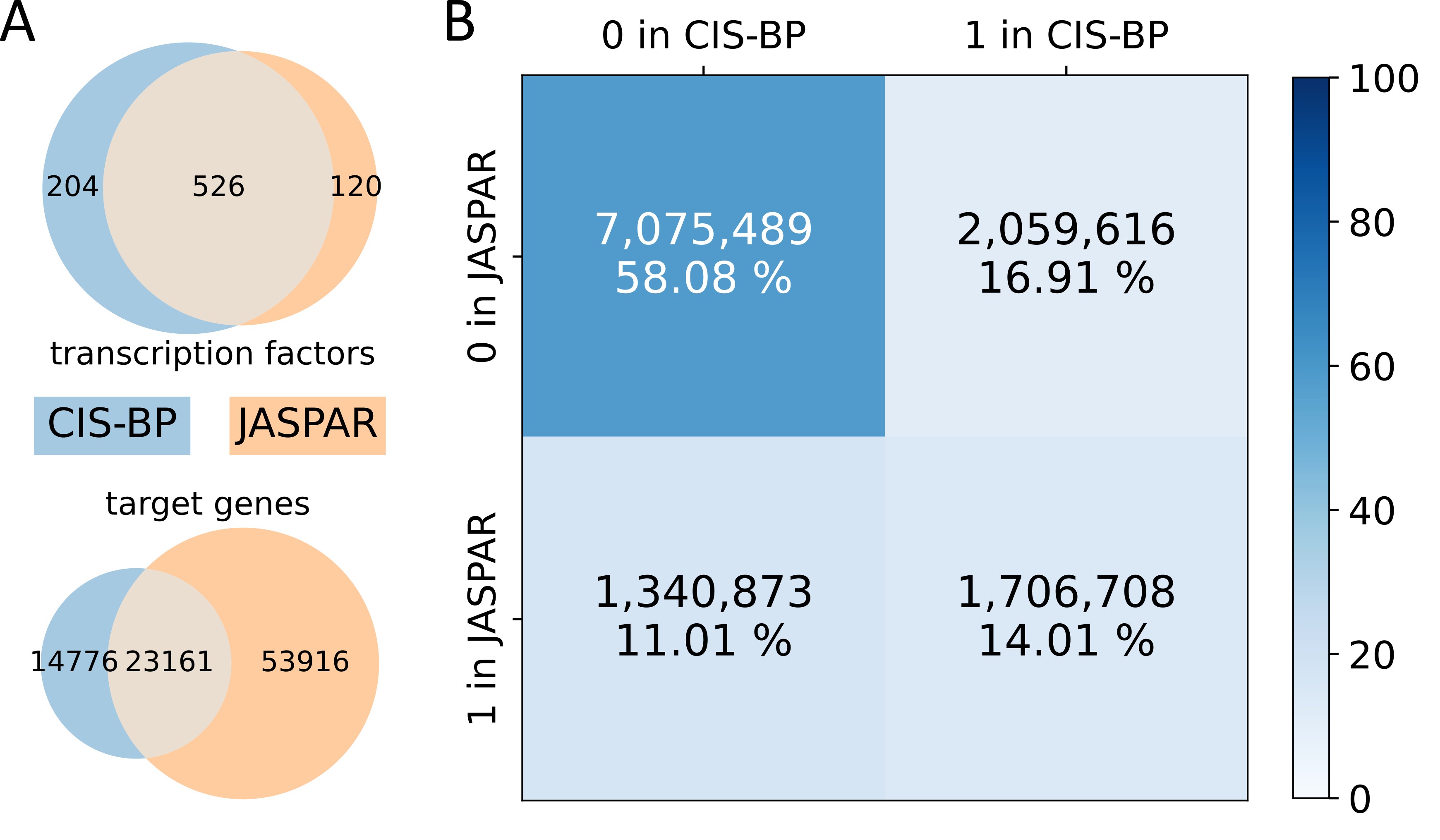

Supplement: btaf320_Supplementary_Data [file btaf320_supplementary_data.zip › figure_s2.jpg]
